# Application of mixed reality technology in talocalcaneal coalition resection

**DOI:** 10.3389/fsurg.2022.1084365

**Published:** 2023-01-06

**Authors:** Jieyuan Zhang, Cheng Wang, Xueqian Li, Shaoling Fu, Wenqi Gu, Zhongmin Shi

**Affiliations:** ^1^ Department of Orthopedic Surgery, Shanghai Sixth People’s Hospital, Shanghai, China; ^2^ Department of Orthopedic Surgery, Shanghai Sixth People’s Hospital East Campus, Shanghai, China

**Keywords:** mixed reality, talocalcaneal coalitions, resection, navigation, computed tomography

## Abstract

**Objectives:**

With positive outcomes recorded, the mixed reality (MR) technology has lately become popular in orthopedic surgery. However, there are few studies that specifically address the utility of MR in talocalcaneal coalitions (TCC) resection. Our goal in this retrospective study is to assess certain data while examining the viability of using MR to treat TCC resection.

**Methods:**

Six consecutive patients with TCC diagnosed by computed tomography (CT) for which nonoperative therapy had failed and MR system assisted TCC resection were included in this study from March 2021 to December 2021. The feasibility and accuracy of TCC resection were assessed by post-operation radiography. The American Orthopaedic Foot & Ankle Society (AOFAS) hindfoot score and visual analog scale (VAS) score were used to assess the recovery condition and pain level pre- and post-operation.

**Results:**

The surgeon can accurately resect the TCC according to the preoperatively determined range by superimposing the holographic model with the actual anatomy of the TCC using an MR system. Additionally, no additional x-ray was necessary while operating. Mean follow-up was 10.3 months, with a minimum of 6 months. There is a significant difference between the preoperative AOFAS score of 53.4 ± 3.8 and the 6-month follow-up AOFAS score of 97.3 ± 2.2 (*p* < 0.05). There is also a significant difference between the preoperative VAS score of 8.1 ± 0.7 and the 6-month follow-up VAS score of 1.7 ± 0.4 (*p* < 0.05). All individuals had clinical subtalar mobility without stiffness following surgery.

**Conclusion:**

While the TCC resection operation is being performed, the application of MR technology is practicable, effective, and radiation-free, giving surgeons satisfactory support.

## Introduction

The most frequent kind of tarsal coalition that causes hindfoot pain is talocalcaneal coalitions (TCC), which is characterized by complaints of flatfeet, foot or ankle pain following mild damage, or recurring ankle sprains (1, 2). According to reports, TCC occurs between 1% and 6% of the time (3). CT scanning continues to be the standard imaging technique for diagnosis and classification (4, 5). Nonoperative treatment, hindfoot arthrodesis, and coalition resection are among the available therapeutic alternatives. Recent research has recommended resection because it preserves motion, leads to better results and has fewer problems, particularly in patients with neutral hindfoot alignment, some subtalar joint motion preservation, and no adjacent joint arthrosis (6, 7).

Recently, orthopedic surgery has made extensive use of digital techniques. Surgical accuracy has been increased with the help of clinical real-time location and visualization made possible by such methods. Following preoperative planning based on CT 3-dimensional (3D) reconstruction, the customized 3D printed surgical guide allows for intraoperative time savings, improved excision accuracy, and decreased exposure to radiation during TCC resection (8). TCC resection has also been performed using computer navigation systems based on fluoroscopy and intraoperative CT guidance (9). Although these methods increased the safety and accuracy of the TCC resection, the intrinsic spatial and temporal separation issue still presents a difficult task for orthopedic surgeons.

In 3D applications, MR technology combines virtual reality (VR) and augmented reality (AR) as a new generation of reality technology (10). The core function of MR technology is to integrate 3D holograms into the users’ perception of the actual world and create an interactive feedback loop between it and the virtual world to improve the sensation of reality and space (11, 12). The integration of MR technology in orthopedic surgery reduces the reliance on the experience of the surgeons, provides customized 3D visualization models for precise diagnosis and treatment of orthopedic abnormalities, and resolves vision deficiencies during the process of synchronizing virtual images and real operative sights (13, 14). The aim of this study was to analyze the integration of the MR navigation system in TCC resection to determine whether it is practicable and effective during the treatment of TCC. We expect this technology to be one of the prominent future development directions in orthopedic surgery.

## Methods

### Study design and participants

From March 2021 to December 2021, 6 patients diagnosed with TCC who did not respond to conservative treatment such as activity modification, foot orthoses, nonsteroidal anti-inflammatory drugs, physical therapy, and cast immobilization were prospectively enrolled into this study. The TCC patients underwent TCC resection by MR navigation. The protocol for this study was approved by the ethics committee of our institution, and written consent was obtained from all participants. Inclusion criteria were symptomatic TCC patients. Excluded criteria were as follows: (1) subtalar joint arthritis, (2) association with another coalition, (3) hindfoot valgus producing lateral impingement.

### Intraoperative guidance and surgical treatment

**Figure 1:**
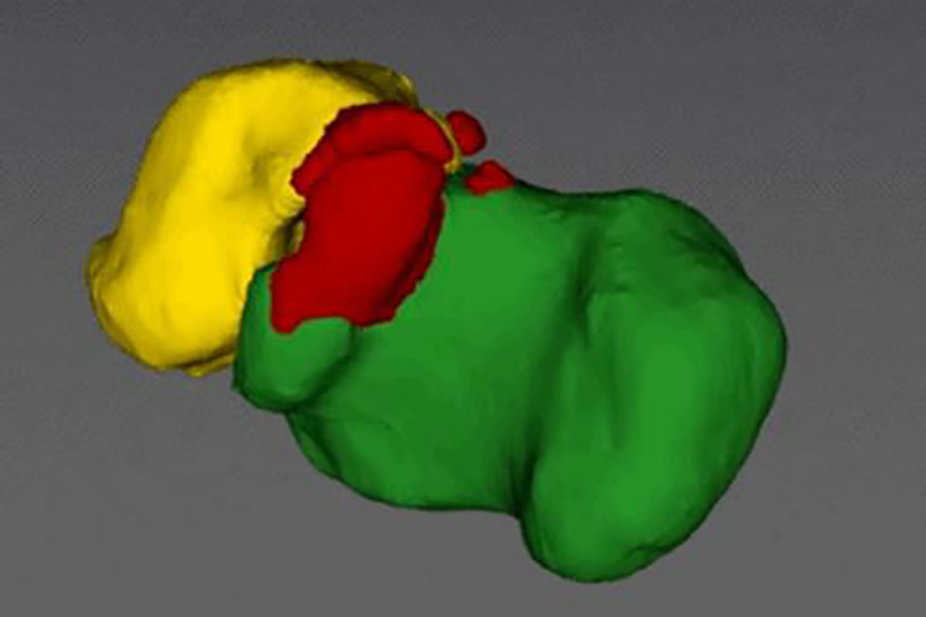
The Arigin3D-STS-Design software intelligently identified the TCC.

CT scans (64 detector rows, 0.625-mm-thick slices, 120 kV, 125 mA, Siemens, German) of each patient's affected side subtalar joint were performed before and after open TCC resection surgery, and the resulting Digital Imaging and Communications in Medicine (DICOM) data were used. To create a “3D subtalar joint digital model,” different colors were employed to distinguish distinct structures. Then, using artificial intelligence on this model in the Arigin3D-STS-Design software (Shanghai Xinjian Medical Technology Co., China), the coalition parts that need to be resected during the operation were identified (Figure 1). It was possible to see the subtalar joint from various angles. The subtalar joint was properly visualized in stereoscopic and apparent views. The HoloLicyo headset was used to create holograms after the 3D model was reconstructed.

**Figure 2:**
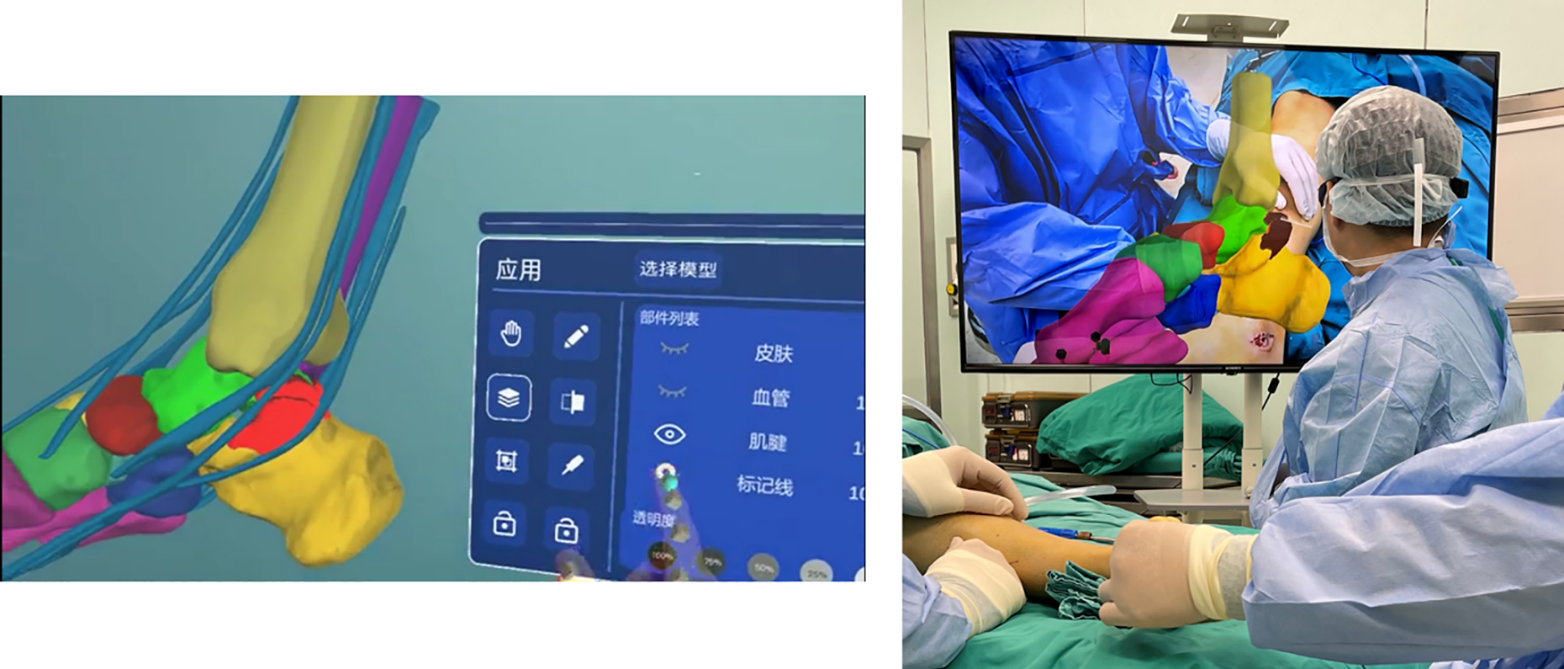
Application of MR-navigation in the TCC resection surgery.

All patients were treated by the same senior surgeon. The surgeons wear HoloLicyo glasses (Shanghai Laiqiu Medical Technology Co., China) while performing surgery so they may interact with the patient's hologram in real time through the MR system (Figure 2). Over the coalition-caused bony hump, and running the full length of the subtalar joint, a medial incision was made inferior to the medial malleolus. An incision is done to open the sheaths of the flexor digitorum longus (FDL) and tibialis posterior (TP). The three bone markers chosen prior to the operation were used to manually rigidly coregister the holograms with the subtalar joint. The resection of the coalition was carried out in accordance with the surgically specified optimal range under the direction of the MR hologram. The posterior facet's normal-looking cartilage was then seen. To further guarantee a total coalition resection, hindfoot mobility wasa evaluated intraoperatively.

One day following surgery, complete weightbearing with full range of motion (ROM) was permitted. After suture removal, active and passive ROM exercises as well as strengthening activities were begun. The patient was permitted to resume regular daily and sporting activities 6 weeks after surgery.

### Evaluation index

The accuracy and safety of the TCC resection were assessed through the postsurgery CT. The postoperative model was also superimposed onto the preoperative model using the ICP (iterative closest point) algorithm.

The American Orthopaedic Foot & Ankle Society (AOFAS) hindfoot score and visual analog scale (VAS) score were used to assess the recovery condition and pain level pre- and post-operation. The subtalar mobility was also examined.

### Statistical analysis

The statistical software SPSS Version 19.0 (IBM, Chicago, IL, USA) was used for the analysis. The Wilcoxon rank sum test was used to evaluate nonparametric data; *p* < 0.05 was considered statistically significant.

## Results

A total of 6 patients were included in this study. There were 3 males and 3 females. The average age of the patients was 22.5 ± 3.1 years. They all possess unilateral CTT. Three patients were determined to have Type I TCC, one patient to have Type II, one patient to have Type III, and one patient to have Type IV TCC (15). All of them underwent TCC resection surgery with the assistance of MR navigation, which took an average of 5–10 min to adjust the hologram to the anatomy, and the complete procedure took an average of 25–40 min.

**Figure 3:**
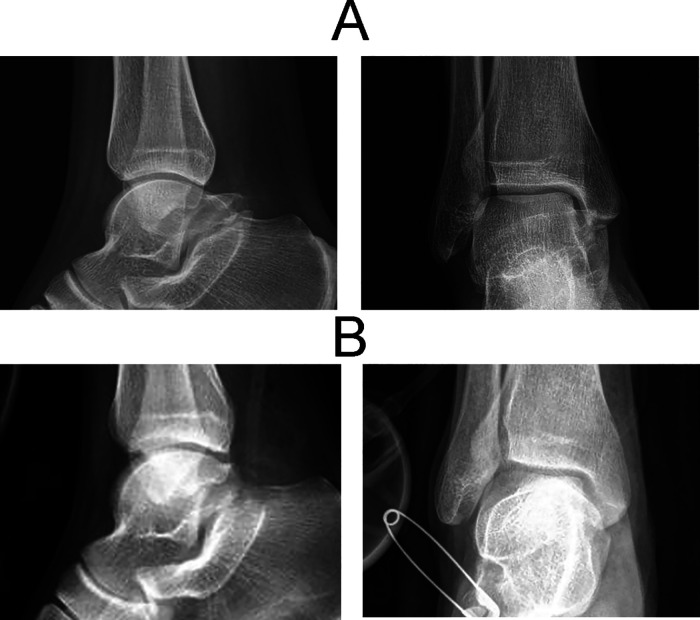
X-rays of TCC pre- (**A**) and post-operation (**B**).

**Figure 4:**
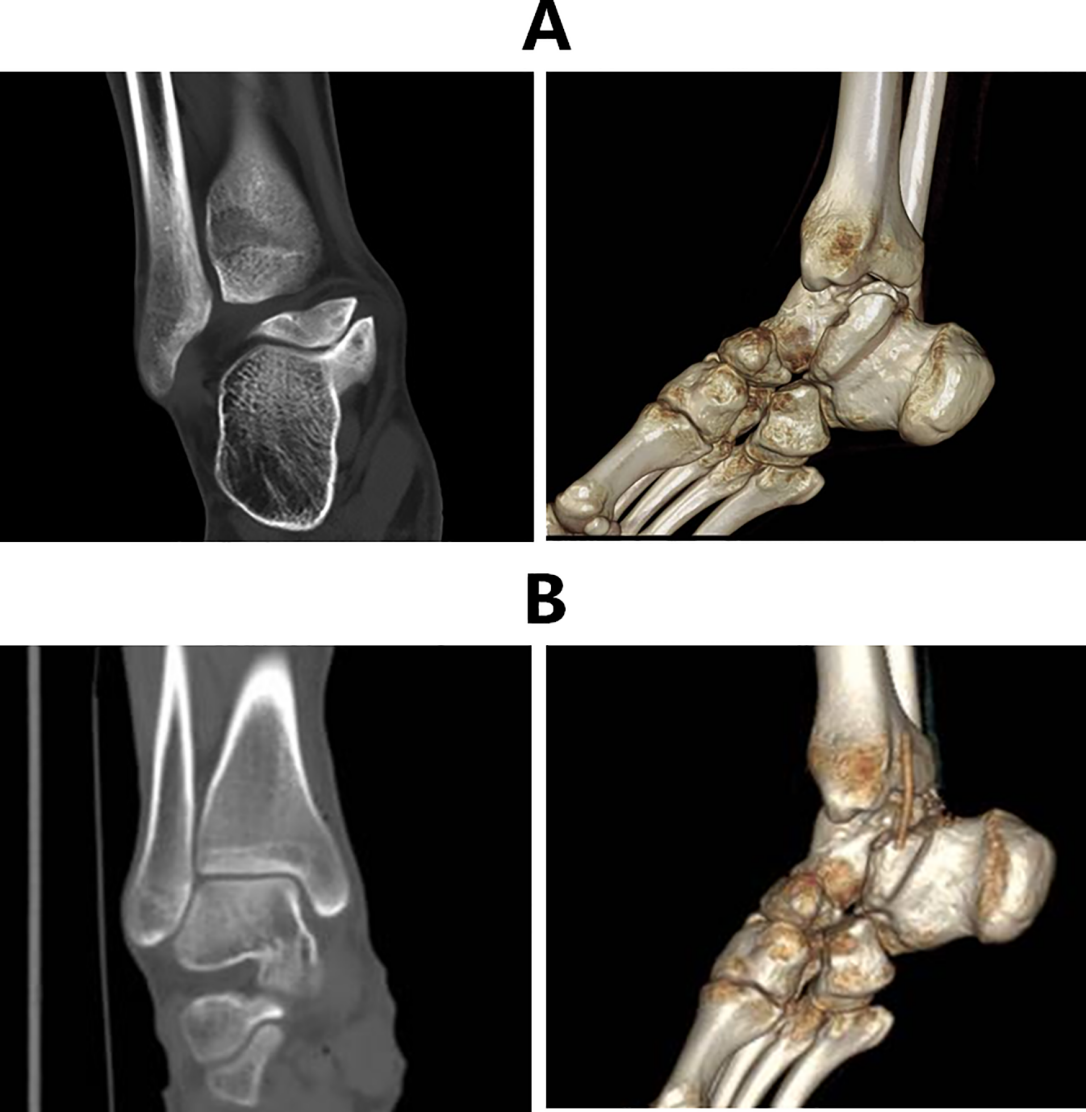
CT of TCC pre- (**A**) and post-operation (**B**).

**Figure 5:**
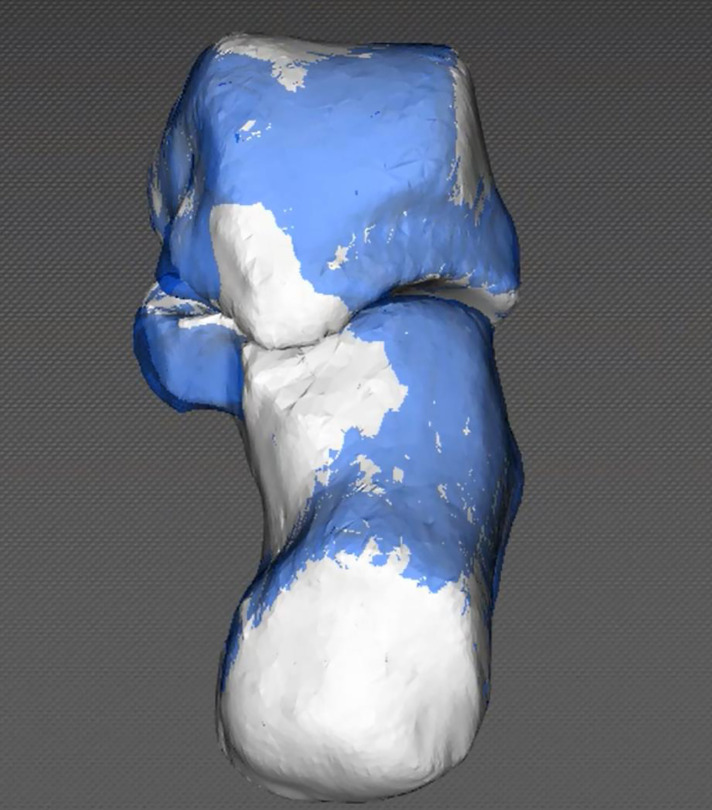
Visualization of the morphologic changes in the postoperative model relative to the preoperative model. The postoperative model was superimposed onto the preoperative model.

The postoperative x-ray and CT revealed that the TCC coalition disappeared (Figures 3, 4). The superposition of preoperative and postoperative models confirmed that the TCCs of all patients were resected safely and precisely according to their preoperatively identified range (Figure 5). Notably, it was not necessary for the radiographic to provide extra TCC coalition information during the operation with the use of MR navigation.

Mean follow-up was 10.3 months, with a minimum of 6 months. The preoperative AOFAS hindfoot score was 53.4 ± 3.8, and the AOFAS hindfoot score was 97.3 ± 2.2 at the 6-month follow-up (*p* < 0.05). The preoperative VAS score was 8.1 ± 0.7, and the VAS score was 1.7 ± 0.4 at the 6-month follow-up (*p* < 0.05).

All of the patients had clinical subtalar motion without stiffness after surgery, whereas none of them had any subtalar mobility before.

After surgery, one patient experienced hyperesthesia over the medial aspect of the calcaneus, but after 3 months, the condition totally resolved.

## Discussion

It has long been known that TCC can cause pain and impairment in young individuals. On the basis of early negative perceptions of resection, traditional treatment suggestions for symptomatic TCC centered on subtalar arthrodesis (16–18). However, the majority of the patient populations in these early publications were elderly patients with different stages of secondary arthritis, which reduced the value of resection. Over time, numerous surgeons showed that removing the affected tissue and inserting an autogenous graft made of fat, extensor digitorum brevis, or split flexor hallucis longus tendons produced positive clinical outcomes (6, 19). Despite these encouraging outcomes, others disagreed with the necessity of resecting TCC encompassing more than 50% of the subtalar joint or those accompanied by valgus deformity (20, 21). However, some people have noted success in treating patients whose coalition involved more than 50% of the subtalar joint (22, 7).

There is now widespread agreement that the best course of treatment for middle facet TCC that continue to hurt is resection and interposition fat grafting. With a mean follow-up of 11.5 years, McCormack et al. looked at 9 symptomatic TCCs that had full resection with fat-graft interposition. Regardless of the percentage of involvement, they advised excision of the middle facet of a continuously symptomatic coalition if the patient showed no signs of degenerative alterations (23). Additionally, Luhmann et al. published their symptomatic TCC indications and outcomes. After analyzing the findings from their 25 coalitions, they advised treating all young patients with symptomatic TCC who had failed nonoperative therapy and did not have an arthritic hind foot with a TCC excision rather than an arthrodesis. They added that patients with TCC higher than 50% or heel valgus higher than 21 might still experience very positive results (22). Notably, a triple arthrodesis is a viable salvage treatment for elderly patients with degenerative alterations or those who have failed a prior resection. However, according to the literature, this has a 20-year lifespan before developing ankle arthritis. To sum up, resection is therefore our recommended course of treatment for individuals with a symptomatic TCC when compared to arthrodesis. TCC resection can be carried out using either the modern endoscopic techniques or the conventional open medial approach. However, the majority of these strategies are only applicable to coalitions that are axially parallel and linear in nature. After preoperative planning based on CT 3D reconstruction has been presented, the customized 3D printed surgical guide has been created (8). The major benefits of the technique tip include reduced x-ray exposure, improved excision accuracy, and reduced intraoperative time. However, in addition to the lack of technological know-how and training among orthopedic surgeons, the primary obstacle to performing this treatment may be the unavailability of a 3D printer, which is currently unusual in most institutions. The use of an intraoperative CT and a navigated instrument system has also been documented (9). This method provides for an effective, complete, and controlled resection, as well as minimizes morbidity because less bone is removed and entire subtalar articulations are preserved but has drawbacks like as radiation exposure and higher expense.

The most current advancement in reality technology is MR. MR provides the user with a sense of depth and perspective, in contrast to AR and VR, which considerably enhances the 3D visualization of surgical anatomy. By anchoring them in the actual world, it also makes it possible for the user to interact with both real and virtual items. As an assist in surgical operations, MR holograms provide a number of benefits. Specifically, real-time 3D holographic sharing, depth fitting between the real world and the virtual world, and real-time interactivity. There are few systematic reviews and research on the use of MR technology in orthopaedic surgery, despite claims of its utility in a number of medical operations. However, the MR-assisted technique has increasingly been employed successfully in recent years for surgeries such placing cervical pedicle screws (24), lumbar fracture (25), total elbow arthroplasty (26), revision hip arthroplasty (27), and knee replacement surgery (28). According to the preoperative strategy, TCC in symptomatic TCC patients was safely and precisely removed in the current study using MR navigation technology. The subtalar joint might be seen at a number of angles. Both stereoscopic and apparent views allowed for a clear visualization of the subtalar joint. After the 3D model was rebuilt, holograms were produced using the HoloLicyo headset. The holograms were manually rigidly coregister with the subtalar joint using the three bone markers that were predetermined prior to the procedure. The fact that the surgeon was wearing MR glasses the entire time had no impact on the procedure. x-ray and CT scans taken after surgery showed no signs of residual coalition. Following surgery, all patients showed clear pain relief on the VAS and functional recovery on the AOFAS (*p* < 0.05). The subtalar mobility was also fully recovered without stiffness, demonstrating the viability, safety, and accuracy of the MR-navigation approach for the treatment of TCC. Short-term follow-up revealed no significant complications. Moreover, no further x-ray films were necessary during surgery. Satisfactory clinical results were also obtained with MR-navigation in one patient whose coalition involved more than 50% of the subtalar joint, and no subtalar joint instability occurred. Confirming our recommendation that TCC resection should be preferred over arthrodesis. This article is the first to describe the application of the MR-navigation technique in TCC excision, to the best of our knowledge. We provide a detailed account of our initial preoperative planning, intraoperative navigation, and surgical experience. Notably, the MR-navigation system is an adjunctive aid, much like other image-guided techniques, and does not, however, replace the anatomical expertise and experience of the surgeons.

However, it is important to point out some restrictions on the use of MR in TCC resection. First, there is need for improvement in registration accuracy. Second, the blending of real-world and virtual images may cause ocular discomfort. Thirdly, there is a need to enhance human-computer connection. Fourth, the results need to be confirmed in randomized studies with more cases because this is a tiny case study without controls.

## Conclusion

The use of MR-navigation technology in TCC resection surgery is practicable, effective, and radiation-free, maybe one of the prominent future development directions in orthopedic surgery.

## Data Availability

The original contributions presented in the study are included in the article/Supplementary Material, further inquiries can be directed to the corresponding authors.
